# Action of Lovastatin (Mevinolin) on an *in vitro *model of angiogenesis and its co-culture with malignant melanoma cell lines

**DOI:** 10.1186/1475-2867-6-9

**Published:** 2006-03-30

**Authors:** Ivan Depasquale, Denys N Wheatley

**Affiliations:** 1 Department of Plastic Surgery, St John's Hospital, Howden Road West, Livingston, West Lothian, EH54 6PP, UK; 2 BioMedES, Leggat House, Keithhall, Inverurie, Aberdeen AB51 0LX, UK

## Abstract

**Background:**

Lovastatin and other statins may reduce the development of melanomas. The effects on melanoma cells and their ability to enhance angiogenesis in a co-culture system presented an opportunity to assess whether Lovastatin act on melanoma cells, HUVEC or both types of cells.

**Results:**

Direct effects of co-culturing two different malignant melanoma cells (A375 and G361) on the process of angiogenesis *in vitro *was studied with our angiogenesis model[1], based on human dermal fibroblasts and human umbilical vein endothelial cells (HUVEC). Co-cultures were set up using "sland" and "dispersed seeding" techniques. A statistically significant increase in tubule formation in both cases was observed compared to controls. The effects of doses equivalent to therapeutic concentrations of Lovastatin were analysed. The drug inhibited the growth of all cell types, induced apoptosis, and markedly reduced the formation of tubules in the angiogenesis model at low concentrations. Its action was successfully reversed by the introduction of geranylgeranyl pyrophosphate.

**Conclusion:**

Lovastatin can reduce both tumour (melanoma) cell growth, and the angiogenic activity of these cells in co-cultures using an established 2-dimensional model angiogenesis system beyond that which would be seen by reduced proliferation alone.

## Background

Malignant melanoma, like most other tumours, requires angiogenesis to sustain growth beyond a critical volume of 1–2 mm, when the diffusional exchange of nutrients and metabolites is exceeded [1-3]. Evidence that the degree of angiogenesis increases with tumour progression in melanoma has recently accumulated [4-9]. Since melanomas tend to invade aggressively from a small size, they are thought to have potent angiogenic stimulating activity.

In a study of a transplantable mouse mammary adenocarcinoma, Thompson *et al*. [2] showed that vascular density within tumours was invariably greater at the periphery than the centre, and that the most pronounced increase in vascular density affected not the tumour so much as the host tissue (stroma) surrounding it, speculating that tumours acquire their vasculature by infiltration into, and expansion between, a network of newly formed vessels in the local connective tissue. It remains unclear how malignant cells orchestrate the process of angiogenesis to maintain the tumour colony and facilitate metastasis. However in the largest study to date on primary malignant melanoma specimens, Kashani-Sabet *et al*. [10] showed that tumour vascularity, assessed by a histological grading score, was the most important determinant of overall (reduced) survival, surpassing tumour thickness.

We asked whether melanoma cells are chemotactically attracted to pre-existing host vasculature in the dermis, where they induce angiogenesis that becomes incorporated into the advancing tumour front; or whether tumour cells induce the migration of endothelial cells that express a pro-angiogenic phenotype into the expanding tumour mass. In an attempt at trying to understand this interaction between malignant melanoma cells and the associated angiogenic vessels, two malignant melanoma cell lines were co-cultured with an angiogenesis model utilising human umbilical vein endothelial cells (HUVEC) and human diploid fibroblasts (HDF). This model generates tubular structures comprised of endothelial cells that have a clear polarity between the luminal and abluminal surfaces, and are surrounded by a thin, partly formed collagen based basement membrane generated predominantly by the HDF [11]. The first part of the study assesses the morphological changes induced in the angiogenic tubules by co-culture of malignant melanoma cells when compared to the angiogenesis model alone.

This angiogenesis model is particularly suitable for analysing a drug's potential for modulating angiogenesis. Lovastatin is a potent inhibitor of hydroxymethylglutaryl co-enzyme A (HMG-CoA) reductase, the rate-limiting enzyme in cholesterol biosynthesis, and an early member of a generation of blood-lipid lowering drugs. HMG-CoA is necessary for the formation of geranylgeranyl pyrophosphate (GGPP) by the conjugation of farnesyl pyrophosphate and isopentenyl pyrophosphate. A study performed by the Air Force/Texas Coronary Atherosclerosis Prevention Trial that evaluated the efficacy of Lovastatin in the prevention of coronary events had highlighted the effects of Lovastatin in the biology of melanoma. In this report, Splichal *et al*. [12] reported a remarkable 48% decrease in the incidence of new melanomas in the treated group (n = 3304 participants) compared to the placebo group (n = 3301). Lovastatin exerts a synergistic anti-tumour activity in the mice melanoma model when used with immune response modifiers, such as TNF-α [13], or when used in combination with cisplatin [14]. Thus evidence is accumulating that Lovastatin, both alone and when used in combination with chemotherapeutic agents, inhibits the progression of melanoma. There have been strong confirmation of this is the work of Li et al. [15], and more recently by Shellman et al. [16] during the time our work was in progress.

The aim of the second part of the study was to assess the effects of exposing the melanoma cell lines in the angiogenesis model as a co-culture to Lovastatin *in vitro*, and to examine the potential role of GGPP.

## Materials and methods

### Melanoma cell line culture

Two commercially available human melanoma cell lines were used. These were:

• **A375 **cell line derived from a 54 year-old female with melanoma, and was supplied frozen by ATCC^® ^(Rockville Pike, MD, USA) Cells of this line display a metastatic phenotype.

• **G361**, also supplied by ATCC^®^, is a malignant melanoma cell line derived from a 31 year-old male Caucasian that produces melanin. This cell type also has a metastatic phenotype.

Both were grown in Dulbecco's modified Eagle's medium (DMEM Sigma^® ^D-5671), to which 5 ml 200 mM L-glutamine, 5 ml 100 mM sodium pyruvate, 5 ml 500× antibiotic/antimycotic solution (Sigma A-9909) and 50 ml fetal calf serum were added per 500 ml. The cells were cultured in 20 ml medium in 75 ml plastic flasks, incubated at 37°C in 5% CO_2 _in air humidified atmosphere. The stock population was maintained by splitting cells just prior to confluence, 5 ml 0.25% trypsin solution being used to release the cells from the substratum.

### Angiogenesis cell lines and model

The HDF cells were obtained from adult human skin taken at breast reductions performed in the Plastic Surgery Department, Grampian University Hospitals Trust with full consent of the patients and the local ethical committee. Fibroblast stocks were cultured in 20 ml DMEM (Sigma) in 75 cm^2 ^flasks. The cells were used when sub-confluent before reaching passage 10. HUVEC cells supplied by TCS Cell Works (Botolph Claydon, Buckingham, UK) were cultured in 20 ml EGM-2 (Clonetics/Biowhittaker) in 75 cm^2 ^flasks. The cells are used at sub-confluence up to passage 6. The technique utilised to generate the co-culture model followed that recommended in ref.12 using 24-well plates, at a ratio of 4:1 (HDF: HUVEC) and at seeding densities of 2 × 10^4 ^HDF and 5 × 10^3 ^HUVEC.

### Immunostaining

Endothelial cell specific primary antibodies used to visualise angiogenesis were anti-CD31 (PECAM-1) Ab (DAKO^®^) and anti-MCAM(P1H12) Ab, (CHEMICON^® ^International, Inc), which reacts specifically with CD146. Three different chromogens were used for the immunoassay, namely DAB (brown), Sigma FASTTM BCIP/NBT (purple) and Sigma FASTTM Fast Red TR/Naphthol AS-MX (red). Immunostaining was performed *in situ *in the well plates. Images of all immunostained preparations were captured microscopically with a JVC video camera, coupled to a Neotech Image Grabber/PC, on an Olympus IX 50 microscope.

### Melanoma cell and angiogenesis co-culture

The triple co-culture was performed using 2 techniques, depending on whether the seeding of melanoma cells was performed as an aggregated mass of cells (island) or a dispersed suspension of cells inoculated into wells prior to the addition of HDF and HUVEC suspensions.

#### a) Island technique (G361)

G361 cells were harvested from a 25 cm^2 ^flask, prior to confluence and suspended in 0.5 ml DMEM with its usual additives. They were mixed with HDF cells in a ratio of 2:1 to aid adherence and containment of the melanoma cells into an island. One μL of the suspended cell mixture was carefully pipetted into the centre of each well and left undisturbed for 15 min under the laminar air flow hood to ensure good initial adherence of the clump of cells, and the plate was subsequently transferred to the incubator for 24 h.

#### b) Dispersal technique (A375)

A375 cells were harvested and suspended in 0.5 ml DMEM with its usual additives at densities of 5 × 10^3^, 1 × 10^3 ^and 2 × 10^2 ^cells per well, in a 24-well plate that included controls (no seeding). The suspended cells were gently agitated following deposition in the wells to ensure distribution on the floor of the well before the plate was transferred to the incubator for 24 h.

Following incubation, melanoma cells adhered strongly to the floor of the wells. At this point, the medium was aspirated and 0.5 ml suspension of HDF and HUVEC cells in EGM-2 were gently micropipetted into each well, followed by mild agitation to ensure dispersion of the cells. The co-cultures were incubated for 14 days with medium changes every 2–3 days. Immunostaining was performed on day 15 using anti-MCAM Ab as a marker of endothelium.

### Lovastatin treatment

Lovastatin (Mevinolin, M2147) supplied by Sigma^® ^has an empirical formula of C_24_H_36_O_5_, and is 2-methyl-1,2,3,7,8,8a-hexahydro-3,7-dimethyl-8- [2-(tetrahydro-4-hydroxy-6-oxo-2H-pyran-2-yl)-ethyl]-1-naphthalenyl ester butanoic acid. It is a white crystalline powder, insoluble in water. A stock solution was prepared by dissolving it in 100% ethanol at 12.3 mM. Two different sets of experiments were set up:

#### a) Exposure of the individual cell types

Two 96-well plates were set up with A375, G361, HDF and HUVEC cell suspensions in the appropriate media, 100 μL of cell suspension in each of 46 wells per cell type. Lovastatin was added to each well as 100 μL solution resulting in final 10-point dilutions from 0.097 μM to reach 50 μM in the last set of wells. The set included controls for each cell type where no Lovastatin was added. The plate was incubated for 72 h before the neutral red assay was performed, which is a colorimetric assay based on the uptake of the dye neutral red by viable, active cells devised by Borenfreund *et al*. [17]. The dye localises to the lysosomes and is extracted from the cells with acid solution. The absorbance of the extracted dye was read at 540 nm using a DIAS plate reader (Dynatech Labs^®^), and the calibrated intensity correlates with the number of active cells.

#### b) Exposure in the angiogenesis model

A 24-well plate was prepared with the angiogenesis co-culture as described earlier. Lovastatin was added to each well on day 2 in 50 μL of the stock solution, resulting in final 7-point dilutions from 0.781 μL to 50 μL. The cultures were assayed on day 14, by first fixing and immunostaining, using anti-MCAM Ab and Fast Red TR/Naphthol AS-MX to identify endothelial cells. Quantification of tubule length was performed using a digital imaging technique, by determining the number of pixels in the captured image that correspond to the red stained endothelial cells. Twelve images for each well and control were analysed and the mean calculated for each.

The computer software utilised for this image analysis was AngioSys^® ^Version 1.0, supplied by Adaptix^®^. Images of all immunostained preparations were captured microscopically with a JVC video camera, coupled to an image and microscope, as described above.

### Reversal of Lovastatin with GGPP

GGPP was supplied by Sigma^® ^(G6025) as a solution at 1 mg/mL in methanol: 10 mM aqueous NH_4_OH. Its molecular formula is all trans-3,7,11,15-tetramethyl-2,6,10,14-hexadecatetraenyl pyrophosphate ammonium-potassium salt.

#### (a) A375 reversal

From the results of Lovastatin inhibition of the A375 cell line, 2 sets of 48 well plates with 2 different final reference concentrations of Lovastatin exposure were used, 3 μM and 6 μM. GGPP solution was added to each well in 10-point dilutions from 1.95 μg/ml to reach 100 μg/ml in the last set of wells. The set included controls of cultured cells without Lovastatin or GGPP and with Lovastatin only. Incubation was allowed for 72 h before being assayed using neutral red.

#### (b) HUVEC reversal

The procedure was similar to that applied for the A375 cell line and 2 sets of 46-well plates were set up at final reference concentrations of 3 μM and 6 μM Lovastatin. Incubation continued for 72 h and the wells were assayed using neutral red.

## Results

### Exposure to Lovastatin

#### a) Melanoma and angiogenesis co-culture Island technique (G361)

**Figure 1:**
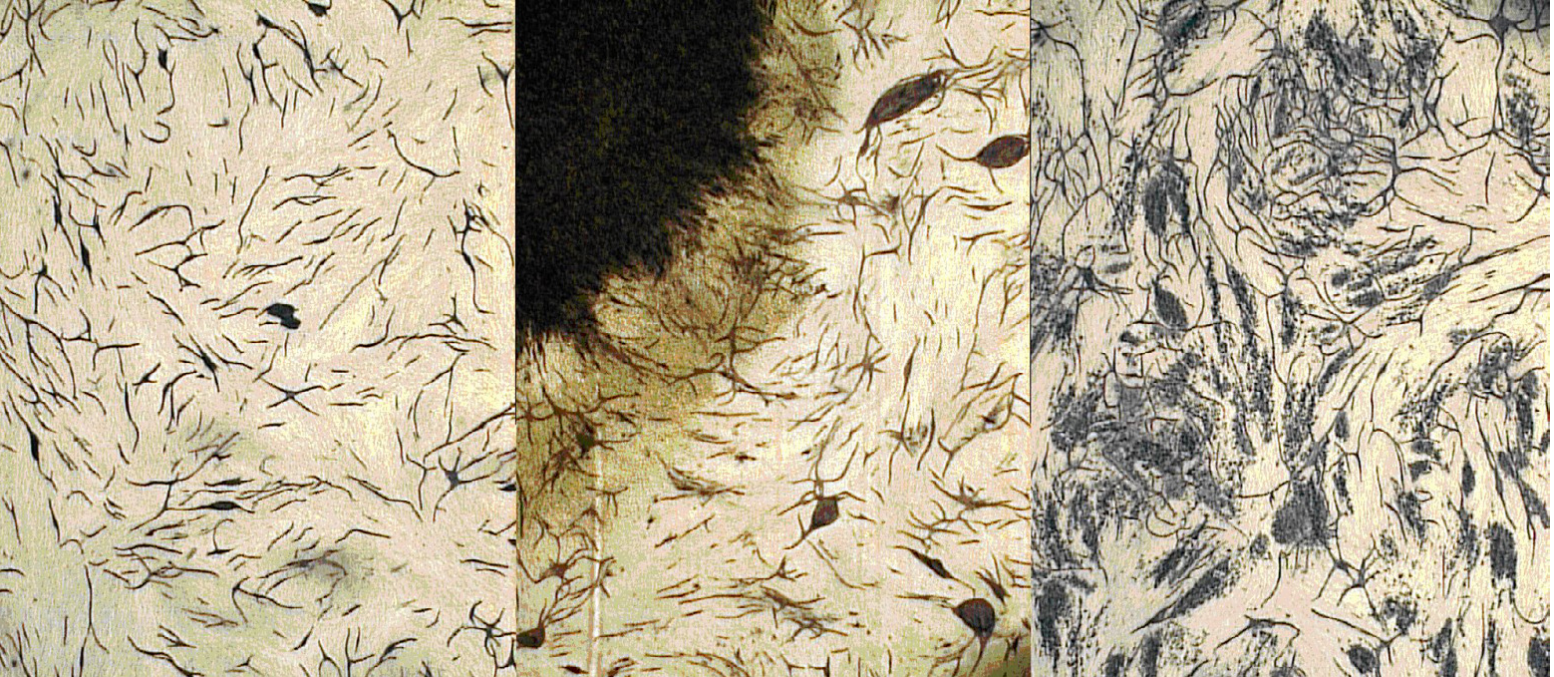
Tubule morphology shown in the different approaches used in angiogenesis model, G361 ("Island") and A375 ("dispersal") co-cultures. Day 14, MCAM stain; 40×. a) Basic angiogenesis model b) Angiogenesis in a G361 co-culture (island) c) Angiogenesis and A375 co-culture (dispersion)

**Figure 2:**
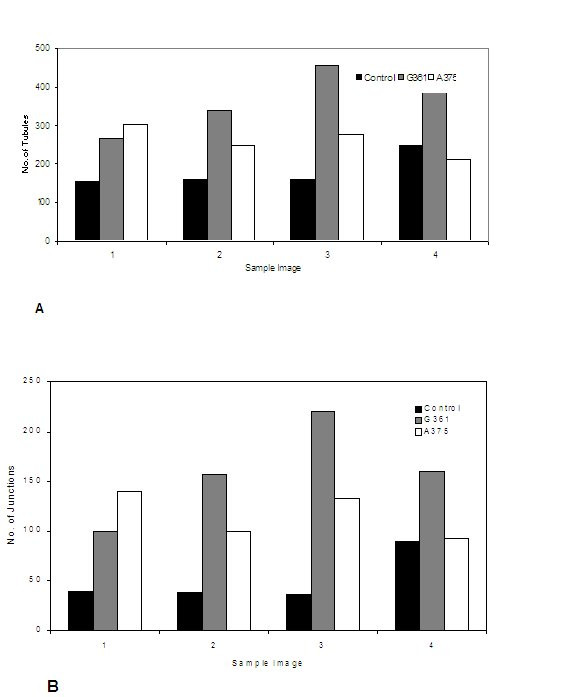
**A: **Number of tubules in 4 sample areas examined for angiogenesis model alone (control), and angiogenesis in G361 and A375 co-cultures. **B **Number of tubule junctions (branching) in 4 sample areas examined for angiogenesis model alone (control), angiogenesis in G361 and A375 co-cultures.

Compared to the controls, the wells containing G361 islands formed denser networks of tubules, assessed visually. The tubules were of the same diameters as in the controls, but were shorter in length and had greater branching (Fig. 1a–c). Quantitative analysis of tubule number and branching was then performed using computer image analysis. The images were taken at 'hot-spots' that included clusters of melanoma cells. The results confirmed ~ 2-fold (201%) increase in the number of tubules and ~ 3-fold (310%) increase in the number of junctions or branches (Fig. 2A; see Discussion).

#### b) Melanoma and angiogenesis co-culture dispersal technique (A375)

Examination under the light microscope revealed that the density of tubules was higher around the melanoma cell clusters and, although branching was increased, the visual impression was that it was distinctly less than that observed for the G361 cell line (Fig. 2B). Quantitative analysis was then performed as above, for convenience sake again at the 'hot spots'. The data confirmed the visual impression, with the number of tubules increasing to 144% and the number of branchings to 227% of the control values.

### Individual cell types

**Figure 3:**
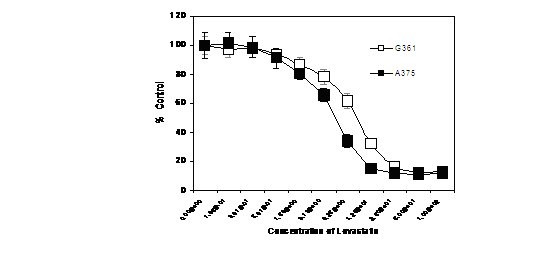
Estimation of percentage of viable melanoma cells (G361 and A375) after incubation with Lovastatin for 72 h, using neutral red assay.

Fig. 3 shows the percentage of A375 cells viable after 72 h exposure to increasing concentrations of Lovastatin, where 100% represents the value for the control, as measured by the neutral red assay. There is an observed incremental loss of viability, starting from the weakest concentration of 0.1 μM, up to a concentration of 6.25 μM, after which the value plateaus at just over 10% viability. The concentration at which 50% viability was achieved (CT50), calculated from the graph, was 3.13 μM. Fig. 3 also has the graph for the neutral red assay with the G361 cell line. A plateau is reached in an analogous way to that for the A375 cell line, but at a higher concentration of Lovastatin (~ 50 μM). The CT50 was 12.5 μM for the G361 cell line.

**Figure 4:**
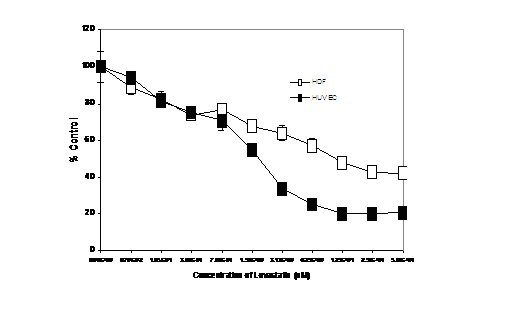
Estimation of percentage of viable HUVEC and HDF after incubation with Lovastatin for 72 h, using neutral red assay.

Fig. 4 is a graph of the neutral red assay for HDF and HUVEC; as seen with the melanoma cell lines, increasing exposures to Lovastatin resulted in an steady decrease in viable cells. The CT50 values for HDF and HUVEC were 11 and 2 μM, respectively.

**Figure 5:**
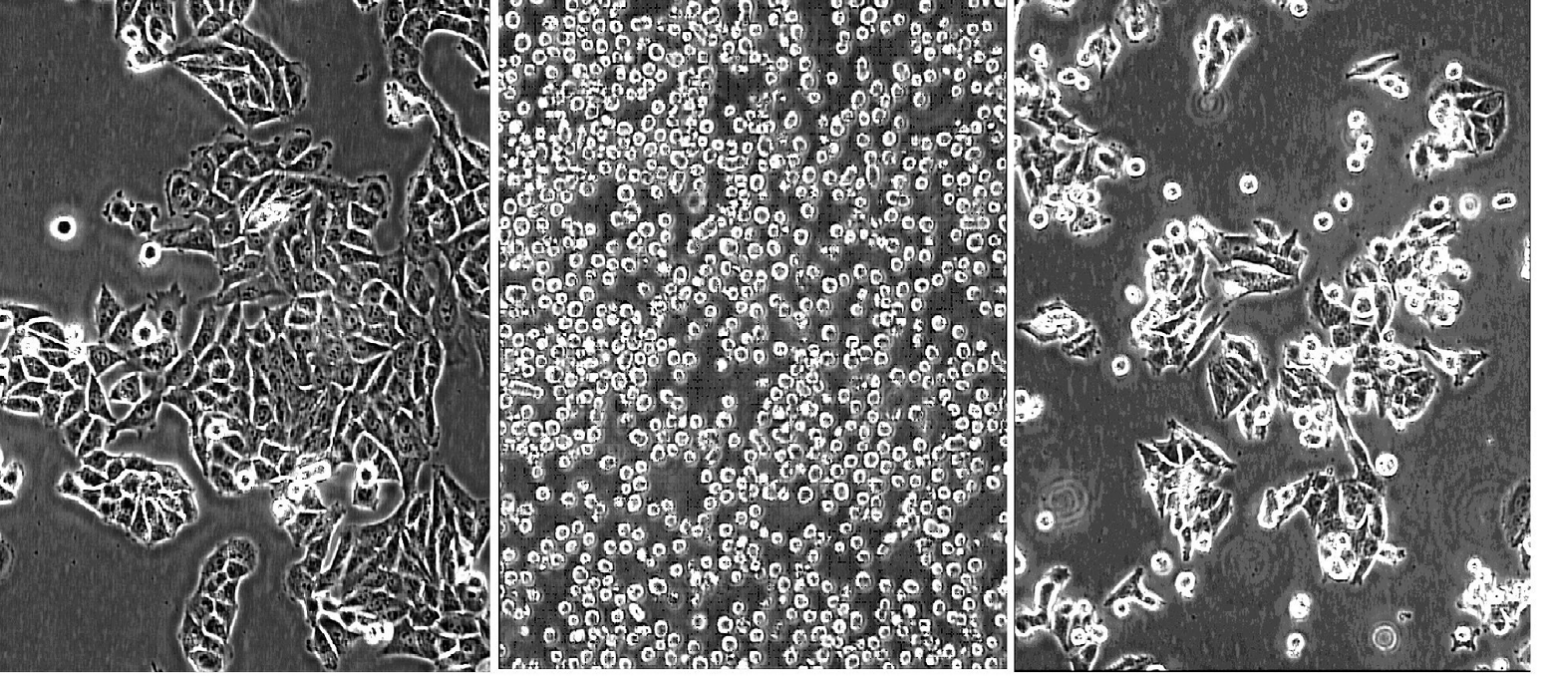
Rounding up of A375 cells following 72 h exposure to Lovastatin and subsequent reversal after change to Lovastatin-free medium. a) DMEM control b) Lovastatin 3 μM c) 72 h recovery in DMEM

Examination of the effect of exposure to Lovastatin on the 4 different cell lines by light microscopy revealed that the cells lost there adherent phenotype and assumed a circular morphology. This is shown here in a representative example of an experiment performed with the A375 cell line (Fig. 5a–c). The cells were exposed to 3 μM (~ CT50) concentration of Lovastatin for 72 h incubation, which demonstrates the cells rounding up. The cells were washed to remove the drug and re-incubated in DMEM with additives for a further 72 h. Evidence of recovery of the adherent phenotype was apparent by a large percentage of the surviving cells regaining their normal morphology and adherence to the substratum.

### The angiogenesis model

**Figure 6:**
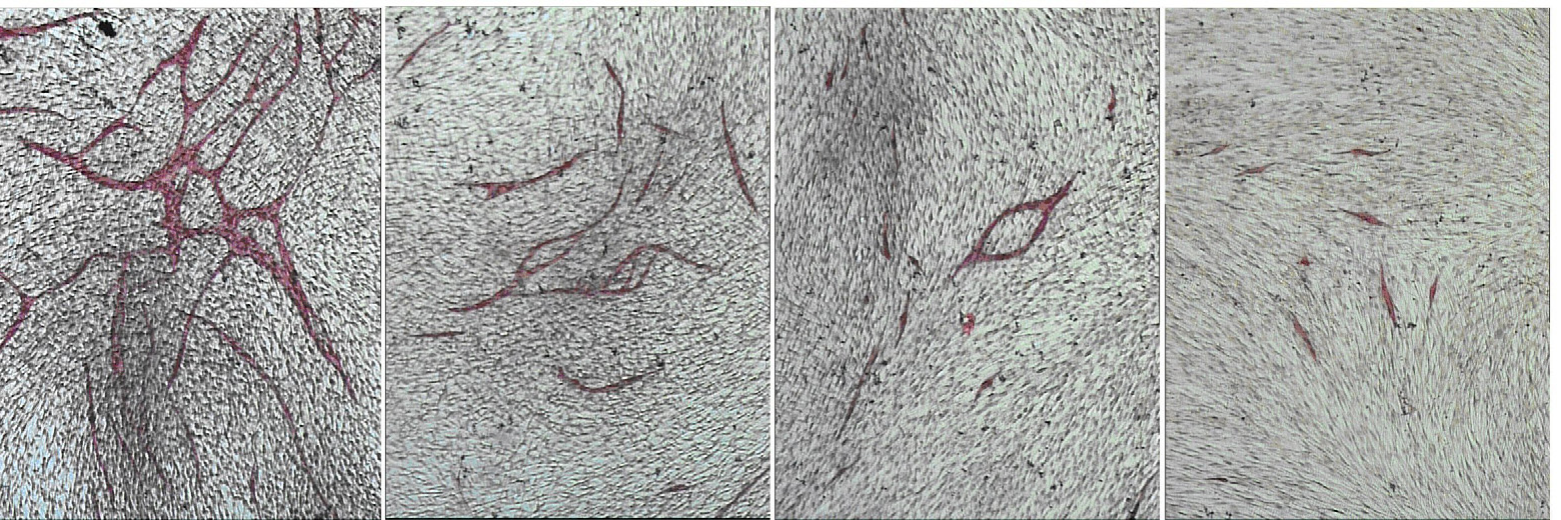
Effect of increasing Lovastatin concentration on angiogenesis development. MCAM staining, 40×. a) Angiogenesis control b) Lovastatin 0.781 μM c) Lovastatin 1.562 Mm d) Lovastatin 3.125 μM

**Figure 7:**
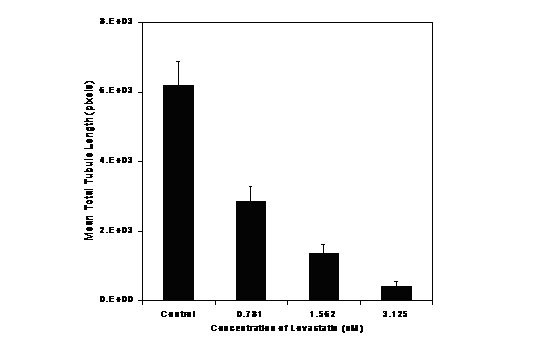
Effect of exposure of angiogenesis model to increasing concentrations of Lovastatin on tubule length.

The observation from the preceding experiments that the CT50 values for HUVEC and HDF were different indicated a different sensitivity to Lovastatin by the two cell lines. In fact HUVEC cells are more sensitive to the effect of Lovastatin (<25% are viable at 6.25 μM). Light microscopy of the angiogenesis cultures exposed to 7 ten-point dilutions of Lovastatin from 0.781 to 50 μM showed that the degree of tubule formation decreased with increasing Lovastatin concentration, and was effectively absent beyond 6.25 μM (Fig. 6). This observation was quantified by computer image analysis and estimations of the mean total tubule length for 3 concentrations (0.781, 1.562 and 3.125 μM) are given in Fig. 7.

### Reversal by GGPP

**Figure 8:**
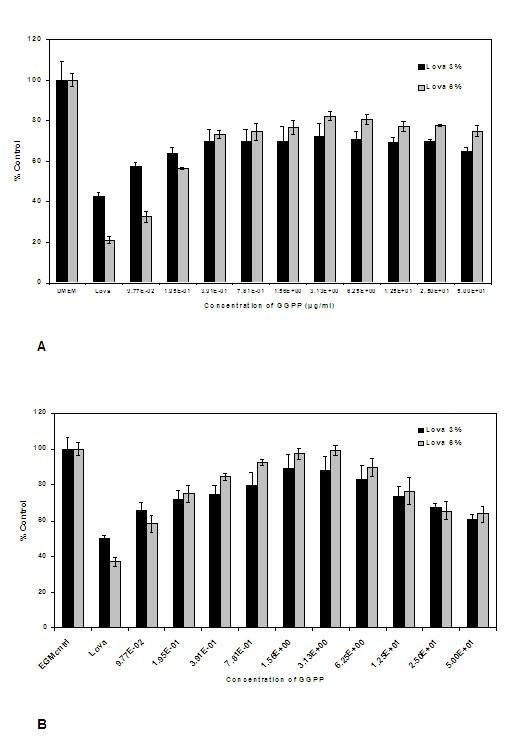
**A: **Estimation of viable melanoma cells (percentage of untreated controls) after incubation with Lovastatin and reversal with increasing concentrations of GGPP. **B: **Estimation of viable HUVEC (percentage of untreated controls) after incubation with Lovastatin and reversal with increasing concentrations of GGPP.

Reversal of the A375 cell line from the effects of Lovastatin was performed on 2 sets of 46-well plates, with 2 different final reference concentrations of Lovastatin (3 and 6μM), and a control. The cultures were incubated for 72 h and assayed with neutral red. As anticipated, increasing concentrations of Lovastatin resulted in a progressively lower percentage of viable cells, as seen in the controls (Mev 3 and 6μM, Fig. 8A). Incubation with increasing concentrations of GGPP reversed the phenomenon up to a maximum of >80% of the control level at a GGPP concentration of ~ 3.1μg/mL for Lovastatin exposures of 3 and 6μM.

Reversal of the HUVEC cell line from the effects of Lovastatin was performed in a similar manner on 2 sets of 46-well plates with the same 2 final reference concentrations of 3 and 6μM Lovastatin, and a control. A similar effect to that observed for the A375 cell line was noted (Fig. 8B). Reversal of the effects of Lovastatin was observed, maximal at a GGPP concentration of 3.13μg/mL. The reversal was even more marked for the HUVEC, which came close to 100% of the control at a Lovastatin concentration of 6.0 μM.

## Discussion

### Melanoma and angiogenesis co-cultures

The characteristics of the angiogenesis model of Bishop *et al*. [11] have proved reproducible and predictable. The sequence of initial fibroblast overgrowth followed by formation of HUVEC clusters, and the subsequent organisation of HUVEC into chord-like structures by days 7–8, followed by their maturation into authentic tubules as a network by days 11–14, are now well-established features. Although reports have come out which have reached similar conclusion to our own on the action of Lovastatin, we deliberately applied it to the Bishop angiogenesis model because this is quite novel relative the more single-cell approaches of most other reports (e.g. 12, 13, 1, 15, 16, 19]. The complex situation requires that we discuss our reasons for the different, often difficult, choices we have made.

To investigate the effects that melanoma cells exert on the developing angiogenesis model, consideration was given to the following. The first related to the physical localization of the melanoma cells relative to the angiogenesis cells. Two main options were available. The Transwell model, which involves keeping the melanoma cells separate by growing in chamber situated superior to the developing angiogenesis model was one possibility. The permeable membrane would allow any released cytokines to influence the co-culture, while the alternative was to grow the cells in direct contact with the co-culture. The advantage of the transwell model is mainly that immunostaining assessment of the vessel growth in the angiogenesis compartment would be easier without the interference in image analysis of the tubules due to co-staining of the melanoma cells by the MCAM antibody (also expressed by A375 and G361 cells). A way around for this problem was to use separate (specific) markers for melanoma, such as HMB45 Ab, and endothelial cells that could be visualised using immunofluorescence technology rather than with chromogens. The limitation of this technique is, however, that any effects due to direct cell contact of the different cell types are going to be abolished, as they would *in vivo*. Another problem was the relative hypoxia found at the interface between the two sets of cells, which can potentially interfere with regular cell growth and proliferation. The alternative was to grow the melanoma cells as a co-culture together with the angiogenesis cells, which seemed the better option and was adopted in our present experiments.

Our next choice was in the timing of the admixing. Considering the situation *in vivo*, where melanoma cells are inducing angiogenesis *in situ *in the dermis of the skin, melanoma cells invade through the ECM of the host tissue and induce a pro-angiogenic influence around them. For angiogenesis to take place, local vasculature must provide ECs, which sprout 'buds' to form new tubules, and this source of EC is available in the form of the resident microcirculation of the dermis. We decided first to seed the melanoma cells in the wells, allow 24 h for adherence, and then add the angiogenesis cells.

A third consideration was the relative proportion of cells to use for the co-culture. The division time for each cell line is specific and varies greatly between cell types, as also between different batches of the same cell type. Fibroblasts grow faster than HUVEC, and if equal cell numbers were seeded in the angiogenesis model, they could quickly overwhelm the HUVEC population, outstripping them of nutritional requirements and compromising the model. For this reason, the seeding of the angiogenesis model is normally performed with a HDF: HUVEC ratio of 1:4 at carefully selected cell densities, which makes all the difference between success and failure. The recommendations for the angiogenesis model suggest that, depending on the characteristics of the particular batch, seeding densities may have to adjusted at each set-up, and ratios of up to 1:8 are not uncommon.

Two further options were considered for the melanoma cell lines. The first was to seed the melanoma cells as a clump or island of cells in the centre of the well. This was achieved by carefully depositing the cells in suspension, as a 1 μL of a mixture of melanoma and HDF cells, in a ratio of 2:1, into the centre of each well and being left undisturbed for 15 min in the laminar air flow hood to allow initial adherence of the clump of cells. The fibroblasts were added to help adherence and containment into an island. The plate was then transferred to the incubator for 24 h, after which the angiogenesis cell mixture was added. The intention was to simulate the tumour mass *in vivo*, which would influence the tubule formation around it, both by the release of cytokines and direct cell contact. The disadvantage of this technique is that cytokines released by melanomas in the centre of the island would probably be diluted by the time they reach the nearest developing tubules and cell-to-cell contact is only available at the periphery of the islands, although this may be a better mimic of the early stages of tumour angiogenesis *in vivo*. The effect on angiogenesis was therefore limited mainly to the area immediately adjacent to the islands.

The other option involved seeding of the melanoma cells as finely dispersed cells along the whole surface of the floor of the well. This was achieved by gently agitating the suspended cells after deposition into the well. Three different seeding densities of melanoma cells per well were tested, 5 × 10^3^, 1 × 10^3 ^and 2 × 10^2^, and the best results were achieved with a density of 1 × 10^3^. Higher seeding densities resulted in an overgrowth of the melanoma cells relative to the angiogenesis cells, which tended to compromise the results. The advantages of this technique are that it allows more direct cell-to-cell contact, it favours the action of melanoma cytokines from most of the tumour cells, and that the effect on angiogenesis can be observed throughout the whole surface of the well. The potential disadvantage is that the co-expression of MCAM by the melanoma cells can interfere with computer assisted, digital quantification of tubule formation. This can be minimised by using digital subtraction techniques, both through manual and software mediated image manipulation to exclude the melanoma cell staining.

The final consideration related to the frequency of medium changes. A balance had to be reached between the replenishment of nutrients and removal of waste metabolites, and the loss of secreted cytokines that would be present in the aspirated medium. The decision was taken to change the medium every 2–3 days, an approach that did not appear to compromise the model.

[We have discussed these problems in detail because they are often crucial to the successful use of the model system and would not have been appropriate in the *Materials and methods *or *Results *sections. For this reason, we have put the former section up front and not after this Discussion. The limitation of any *in vitro *technique is that in trying to simulate the *in vivo *scenario, only a few of the known (an unknown) variables that affect the system in vivo can be "reproduced". One variable that is conspicuous by its absence is the effect that other cell types present in the primary melanoma tumour milieu has on the tumour biology, particularly tumour associated macrophages (which characterise a high proportion of melanomas) and lymphocytes, and the role of pericytes in microvascular biology. Another factor is that a significant proportion of the angiogenic blood vessels *in vivo *are functional (i.e. blood flows in them), which carries many factors capable of modulating the local balance in the development of angiogenesis, and which cannot be reproduced in this model.]

### General outcome

Having listed most of the difficulties in using a model that tries to replicate a *natural set of circumstances*, the results from both models are nevertheless encouraging in that a detectable effect of the melanoma cells on the degree of angiogenesis was evident, notably as an increase in density of tubules, and this was confirmed by quantitative analysis. Using the anti-MCAM Ab to identify endothelial cells, images of the stained co-culture were uploaded into the computer and analysed using specialised software specifically designed for the assessment of angiogenesis tubules. In the case of the island technique (G361), images were captured from the area immediately adjacent to the perimeter of the island. For the dispersal technique (A375), however, a different approach had to be used. The seeded melanoma cells, randomly scattered on the floor of the well, had developed into clumps of cells over the 14 days of incubation with the angiogenesis cells. Tubules appeared to be centred particularly on these clumps, forming hot-spots reminiscent of angiogenic hot-spots observed in primary melanoma specimens. The implication was of an effect of pro-angiogenic stimulation, in close vicinity to the clumps of developing melanoma cells. In the intervening areas between these clumps, angiogenesis was comparable to the control. For these reasons, images taken for tubule analysis were centred about these hot-spots, to represent more accurately the pro-angiogenic potential of the melanoma cell clumps.

Our quantitative data support the hypothesis that the melanoma cell lines exert a pro-angiogenic influence in the model. In the case of the island model (G361), the increase in the number of tubules in the immediate vicinity of the island, was by >200% compared to the control. The morphology of the tubules formed was also different in that they were shorter but more branched, as indicated by the number of tubule junctions which was 310% higher than the control, forming a denser network. These morphological changes progressively became less evident as the distance from the island periphery increased, with a tendency to more closely resemble the control towards the edge of the well. Thus the range of the proangiogenic effect of melanoma could be assessed.

In the case of the dispersal model, the morphologic changes were similar but less marked. The number of tubules was up to 144%, and the branching up to 227%, compared to the control. However, in contrast to the island model, these changes involved most of the surface of the well, reflecting the random distribution of the melanoma cells when seeded on day 1, and only tended to return to control levels in the small areas furthest away from the melanoma cell clumps. Another difference was in the mean tubule length, 45.1 pixels for the dispersed compared to 29.9 pixels in the island model. The mean tubule length in the control model was 41.9 pixels.

Apart from confirming the pro-angiogenic properties of both cell lines, further inferences can be made on the mechanism of this action, by comparing the results obtained from the two sets of experiments. The neoplastic cell lines releases pro-angiogenic factors into the medium, which become progressively more diluted the further the distance from the secretory cell. Because the number and branching of tubules was significantly higher in the case of the island model, it appears that the increase cannot be solely explained by the increased concentration of cytokines produced by the larger number of individual cells forming the island. It could be that the greater number of cells in the island being in direct contact with each other, mediate a higher pro-angiogenic stimulus by positive feedback generated through this contact stimulation, effectively multiplying the pro-angiogenic effect. It also became apparent that "branchings" were sometimes more apparent than real, as will be shown in a following communication. This is because the angiogenesis model being used is more a quasi-3D model with enough depth for tubules to be passing over and under one another rather than truly intersecting in some cases.

The conclusion to be drawn from these experiments is that both the island and dispersal models, as described, represent excellent systems for investigating the angiogenic effects of melanoma (and clearly many other tumour) cell lines. More studies are needed on the model to characterise it further and improve standardisation that will increase its usefulness in the *qualitative and quantitative *analysis of mechanisms involved in the pro-angiogenic activity of melanomas and other tumours.

### Effects of Lovastatin

Jani *et al*. [18] demonstrated that Lovastatin pretreatment of the murine melanoma cells, B16F10, resulted in the inhibition of attachment, motility and invasion *in vitro*. However, evidence of its effect on human melanoma cell lines is not currently available. The aim of this part of the investigation was to determine the effects of exposure of the A375 and G361 cell lines to Lovastatin, together with the cells used for the angiogenesis model both in monoculture and in co-culture. The dilutions of Lovastatin used included the range of 1 to 30 μM, which represents the serum concentration expected in a patient that is using Lovastatin as standard prophylactic treatment of hypercholesterolaemia, and to simulate the conditions in the Air Force/Texas Coronary Atherosclerosis Prevention Trial [12].

The results from the monoculture experiments clearly show that Lovastatin causes a change in the morphology of the cells, causing them to loose their adhesive properties and forcing the cells to assume a circular shape in suspension. In a proportion of cells, this leads to apoptosis and cell death, the degree of which increased as the concentration of Lovastatin increased. Having different CT50 values in the neutral red assay to quantify viable, active cells indicates different sensitivities to Lovastatin. After 72 h exposure to Lovastatin, 50% of the control cells were viable at Lovastatin concentrations of 2, 3.13, 11, and 12.5 μM for HUVEC, A375, HDF and G361, respectively. Thus HUVEC cells were the most sensitive to Lovastatin. As seen from the experiment with A375, the cells that had not become apoptotic did recover the normal morphologic appearance after Lovastatin had been removed and the cells returned to normal medium.

Further evidence of the effects of Lovastatin on cellular function can be seen in the angiogenesis model. The formation of tubules was impaired in a dose-dependent manner. Tubule formation was completely inhibited at concentrations >6.25 μM, and rounding up of the constituent cells was observed at higher titrations. These findings support the results of our *in vivo *experiments (CAM assay) preformed using the Lovastatin analogue, simvastatin, which showed that the drug inhibited VEGF stimulated angiogenesis [19]. Lately we also find that Wei et al. [20] have also looked at the effect of Lovastatin on endothelial permeability – perhaps by a direct interaction with PECAM -1 – which seems to show its inhibition.

The conformational changes of cells treated with Lovastatin reflect the mode of action of the drug. By inhibiting the enzyme HMG-CoA reductase, which is involved in lipid metabolism, the lipids necessary for the normal membrane functioning become defective, and further impairment is seen in their adhesive properties. These are mediated through integrins, which are necessary for cell motility and migration through the ECM in the process of invasion. Furthermore, HMG-CoA has other effects that are independent of cholesterol metabolism, such as the activation of focal adhesion kinase, which plays a role in cytoskeletal reorganisation and consequently cell morphology. Other aspects of the mechanism of action of Lovastatin include the tyrosine phosphorylation of KDR, the VEGF receptor, with implications on angiogenesis that might help explain the experimentally observed inhibition of the angiogenesis model, but these alterations will require specific analyses in future work.

### Reversal by GGPP

One of the modes of action by which the statin group of drugs interferes with angiogenesis is through inhibition of the generation of GGPP generation necessary for the formation of RhoA, and in turn GGPP is a key molecule in angiogenesis. This finding was elegantly demonstrated by Park *et al*. [19], who used the Lovastatin analogue, simvastatin. However, evidence of the importance of this intermediate has not been presented for Lovastatin, which was the aim of our final set of experiments. After A375 and HUVEC had been cultured in medium containing both Lovastatin and GGPP, the metabolite reversed the induction of apoptosis in both cell types in a dose-dependent manner, reaching a peak in both cases at ~ 3.1 μg/mL GGPP. This evidence confirms the role played by Lovastatin in inducing apoptosis, at least partly by inhibition of GGPP formation, with the effect not being limited to neoplastic cells.

### Concluding remarks

Melanoma cells can be grown successfully in angiogenesis co-culture model, which provides a most convenient means for investigations of the effects of drugs and other agents. Particularly significant, however, is the confirmation that Lovastatin inhibits cell growth and induces apoptosis in the A375 and G361 cell lines, which underlines the effects reported in the clinical study of the safety of the use of Lovastatin in the treatment and prophylaxis of hypercholesterolaemia that brought about the observed 48% decrease in incidence of primary melanoma, and continues to support the hopeful prospect that the drug offers some prevention of malignant melanoma.

Both the island and the dispersal technique with the angiogenesis mode have proved useful, and more variations can certainly be used in future, opening up further experimentation into the identity of factors responsible for angiogenesis in melanoma and tumours. It also provides a model to determine the change in expression of markers in the early stages of angiogenesis.

Confirmation of the inhibitory action of Lovastatin on melanoma growth, as demonstrated on the A375 and G361 cell lines, is also encouraging since this was only previously reported in a murine model. Lovastatin has a synergistic effect on melanoma tumour growth in the murine model, when used in combination with other cytotoxic drugs such as cisplatin. However, its action when used in isolation and in the safe therapeutic dose as that used for prophylaxis of hypercholesterolaemia has not been previously demonstrated. The preliminary results show that the action of Lovastatin can be reversed by supplementing GGPP, and therefore clearly indicates at least one mechanism of action.

## Conflicts of interest

The authors declare that they have no conflict of interest, although DNW is Editor-in-Chief of Cancer Cell International, and the work described herein was done under his supervision in his laboratory by ID as a Clinical Research Fellowship towards an MCh degree.

## Authors' contributions

ID carried out much of the experimental work, designed by both authors. DW provided the materials and cultures, along with EB, ready for experimentation and took down much of the data. Much of the data analysis was done by ID.
